# Automated pelvic MRI measurements associated with urinary incontinence for prostate cancer patients undergoing radical prostatectomy

**DOI:** 10.1186/s41747-023-00402-4

**Published:** 2024-01-02

**Authors:** Ingeborg van den Berg, Robert N. Spaans, Frank J. Wessels, Erik J. R. J. van der Hoeven, Charlotte J. Tutein Nolthenius, Roderick C. N. van den Bergh, Jochem R. N. van der Voort van Zyp, Cornelis A. T. van den Berg, Harm H. E. van Melick

**Affiliations:** 1 Department of Radiation Oncology, Division of Imaging & Oncology, University Medical Center Utrecht, Utrecht, The Netherlands; 2 Department of Urology, St. Antonius Hospital, Nieuwegein, Utrecht The Netherlands; 3 Technical Medicine, University of Twente, Enschede, The Netherlands; 4 Department of Radiology, University Medical Center Utrecht, Utrecht, The Netherlands; 5 Department of Radiology, St. Antonius Hospital, Nieuwegein, Utrecht The Netherlands

**Keywords:** Artificial intelligence, Deep learning, Membranous urethral length, Prostate cancer, Urinary incontinence

## Abstract

**Background:**

Pelvic morphological parameters on magnetic resonance imaging (MRI), such as the membranous urethral length (MUL), can predict urinary incontinence after radical prostatectomy but are prone to interobserver disagreement. Our objective was to improve interobserver agreement among radiologists in measuring pelvic parameters using deep learning (DL)-based segmentation of pelvic structures on MRI scans.

**Methods:**

Preoperative MRI was collected from 167 prostate cancer patients undergoing radical prostatectomy within our regional multicentric cohort. Two DL networks (nnU-Net) were trained on coronal and sagittal scans and evaluated on a test cohort using an 80/20% train-test split. Pelvic parameters were manually measured by three abdominal radiologists on raw MRI images and with the use of DL-generated segmentations. Automated measurements were also performed for the pelvic parameters. Interobserver agreement was evaluated using the intraclass correlation coefficient (ICC) and the Bland–Altman plot.

**Results:**

The DL models achieved median Dice similarity coefficient (DSC) values of 0.85–0.97 for coronal structures and 0.87–0.98 for sagittal structures. When radiologists used DL-generated segmentations of pelvic structures, the interobserver agreement for sagittal MUL improved from 0.64 (95% confidence interval 0.28–0.83) to 0.91 (95% CI 0.84–0.95). Furthermore, there was an increase in ICC values for the obturator internus muscle from 0.74 (95% CI 0.42–0.87) to 0.86 (95% CI 0.75–0.92) and for the levator ani muscle from 0.40 (95% CI 0.05–0.66) to 0.61 (95% CI 0.31–0.78).

**Conclusions:**

DL-based automated segmentation of pelvic structures improved interobserver agreement in measuring pelvic parameters on preoperative MRI scans.

**Relevance statement:**

The implementation of deep learning segmentations allows for more consistent measurements of pelvic parameters by radiologists. Standardized measurements are crucial for incorporating these parameters into urinary continence prediction models.

**Key points:**

• DL-generated segmentations improve interobserver agreement for pelvic measurements among radiologists.

• Membranous urethral length measurement improved from substantial to almost perfect agreement.

• Artificial intelligence enhances objective pelvic parameter assessment for continence prediction models.

**Graphical Abstract:**

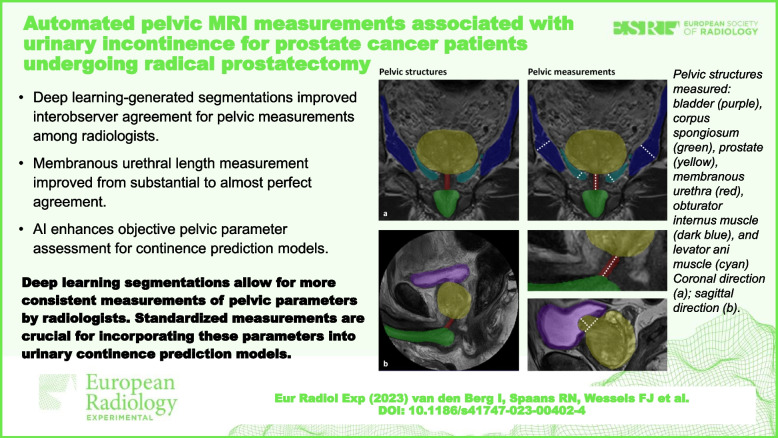

**Supplementary Information:**

The online version contains supplementary material available at 10.1186/s41747-023-00402-4.

## Background

Urinary incontinence is a potential complication in patients with prostate cancer (PCa) undergoing robot-assisted radical prostatectomy (RARP). The 12-month postoperative prevalence of urinary leakage and its interference with daily life were reported by 36% and 17% of men, respectively [1]. Several patient-related variables such as age, body mass index, baseline urinary function, surgical factors, and magnetic resonance imaging (MRI)-based measurements have been found to be associated with post-prostatectomy continence recovery [2–4]. MRI-based measurements include parameters related to the prostate, the urethra, and the pelvic musculoskeletal system, such as the membranous urethral length (MUL), the levator ani muscle (LAM) thickness, and the intravesical prostatic protrusion length (IPPL). Among these measurements, the MUL has demonstrated the highest predictive value, with greater preoperative MUL serving as an independent prognostic factor for continence recovery [2, 5].

The measurement of the MUL is typically performed manually on T2-weighted prostate MRI. It involves determining the distance of the membranous urethra from the inferior border of the prostate apex to the superior border of the penile bulb in either the coronal or sagittal plane. However, manual assessment is prone to considerable interobserver disagreement, with varying intra-class correlation coefficient (ICC) values from 0.37 to 0.57 [6]. To improve interobserver agreement, Veerman et al. [7] investigated the use of standardized anatomical landmarks for MUL measurements, resulting in improved interobserver agreement with ICC values ranging from 0.63 to 0.84 among three radiologists within a single center.

Despite this improvement, Boellaard et al. [8] emphasized the need for artificial intelligence (AI) to further improve interobserver agreement through automated measurements of pelvic parameters. AI models can be used to segment relevant structures and improve the consistency and efficiency of radiologists on prostate MRI [9]. Small deviations in pelvic measurements can have a significant impact on the predictive probability of continence recovery when incorporating them into urinary incontinence prediction models and can influence the treatment choices of PCa patients [10].

Therefore, our study aims to standardize pelvic floor measurements using AI-driven segmentations, enabling automated measurements of the prostate, membranous urethra, and pelvic musculoskeletal structures. We assessed interobserver agreement among radiologists using both manual assessment on raw MRI images and AI-aided visualization of relevant pelvic structures. Additionally, we compared the pelvic measures obtained through the fully automated workflow with those acquired using the manual approach.

## Methods

### Data collection

MRI data for this study was retrospectively extracted from a prospective registry (NCT04228211) where patients had provided informed consent for data sharing. This prospective registry received approval from the institutional review and ethics board of the University Medical Center Utrecht (19–692/M). The study population included 167 PCa patients who underwent RARP within our regional multicentric prospective registry between March 2020 and November 2022. Exclusion criteria included patients with T4 tumors (*n* = 0). We collected preoperative 1.5-T or 3-T prostate MRI data from five Dutch hospitals within our region. Furthermore, MRI scans of three additional patients from three hospitals outside our region were also included in the study. We included the T2-weighted turbo-echo sequences in the coronal and sagittal directions. One sagittal MRI scan had to be excluded due to the presence of motion artifacts. The MRI sequence parameters for each regional hospital are listed in Table 1.

**Table 1:** MRI sequence parameters of coronal and sagittal T2-weighted imaging within our regional muticentric cohort

Sequence parameter	St. Antonius Hospital, Utrecht (*n* = 61)	St. Antonius Hospital, Nieuwegein (*n* = 46)	Diakonessenhuis (*n* = 24)	UMCU (*n* = 17)	Rivierenland (*n* = 8)	Rivas Zorggroep (*n* = 8)
MRI system	Siemens 3 T	Philips 3 T	Siemens 3 T	Philips 3 T	Siemens 1.5 T	Siemens 1.5 T
Scan orientation	Coronal (oblique), sagittal	Coronal (oblique), sagittal	Coronal, sagittal	Coronal, sagittal	Coronal (oblique), sagittal	Coronal, sagittal
TR (ms)						
Coronal	5,000	2,915	7,500	3,000–5,500	4,640	7,500
Sagittal	5,000	4,985	7,400	2,900–5,500	5,060	7,500
TE (ms)						
Coronal	103	95	104	140	108	108
Sagittal	103	95	104	110	87	108
Flip angle (°)						
Coronal	150	90	160	90	160	160
Sagittal	140	90	160	90	150	160
Slice thickness (mm)	3.0	3.0	3.0	3.0	3.0	3.5
Number of average						
Coronal	2	2	3	2	2	3
Sagittal	2	1	2	1	1	3
Number of slices						
Coronal	24	26	25	38	30	20
Sagittal	24	40	25	23	28	24
Reconstructed pixel size (mm × mm)						
Coronal	0.63 × 0.63	0.39 × 0.39	0.27 × 0.27	0.47 × 0.47	0.68 × 0.68	0.63 × 0.63
Sagittal	0.63 × 0.63	0.63 × 0.63	0.27 × 0.27	0.30 × 0.30	0.86 × 0.86	0.63 × 0.63
FOV (mm × mm)						
Coronal	200 × 200	200 × 200	199 × 199	180 × 180	220 × 220	200 × 200
Sagittal	200 × 200	180 × 180	199 × 199	260 × 260	220 × 220	200 × 200

*MRI* Magnetic resonance imaging, *TR* Repetition time, *TE* Echo time, *FOV* Field of view

The following data were extracted from the Utrecht Prostate Cohort database: age at diagnosis, prostate-specific antigen (PSA) at diagnosis, biopsy grade group, Prostate Imaging Data and Reporting System (PI-RADS) version 2 score, clinical stage, and the pathological stage after RARP (Table 2).

**Table 2:** Patient characteristics

Characteristic	Training cohort (*n* = 134)	Testing cohort (*n* = 33)
Age (years), median (IQR)	69 (65–72)	68 (59–72)
PSA (ng/mL), median (IQR)	7.1 (5.1–12.7)	8.1 (5.3–12.9)
Clinical T stage, *n* (%)		
T1	66 (49.3)	18 (54.5)
T2	49 (36.6)	10 (30.3)
T3	17 (12.7)	3 (9.1)
Unknown	2 (1.5)	2 (6.1)
Biopsy Gleason Grade Group, *n* (%)		
1	18 (13.4)	3 (9.1)
2	55 (41.0)	15 (45.5)
3	28 (20.9)	7 (21.2)
≥ 4	33 (24.6)	8 (24.2)
PI-RADS version 2 score, *n* (%)		
2	6 (4.5)	2 (6.1)
3	7 (5.2)	0 (0.0)
4	52 (38.8)	16 (48.5)
5	67 (50.0)	15 (45.5)
Not reported	2 (1.5)	0 (0.0)
D’Amico risk group, *n* (%)		
Low-risk	13 (9.7)	3 (9.1)
Intermediate-risk	70 (52.2)	19 (57.6)
High-risk	51 (38.1)	11 (33.3)
Pathological T stage, *n* (%)		
T2	85 (63.4)	20 (60.6)
T3	48 (35.8)	13 (39.4)
T4	1 (0.7)	0 (0.0)

*PSA* Prostate-specific antigen, *IQR* Interquartile range, *PI-RADS* Prostate Imaging Data and Reporting System

### Pelvic structures

In the coronal plane, the delineated structures encompassed the prostate, corpus spongiosum, membranous urethra, left and right obturator internus muscle (OIM), and the left and right levator ani muscle (LAM). In the sagittal plane, the delineated pelvic structures included the prostate, bladder, corpus spongiosum, and the membranous urethra. The anatomical definition of the pelvic structures was first discussed in a group meeting with three abdominal radiologists (F.W., E.H., and C.T.N., with nine, nine, and seven years of experience, respectively) based on clinical relevance and current literature. The membranous urethra was defined to be delineated from the lower border of the peripheral zone of the prostate to the upper border of the corpus spongiosum. The lumen of the urethra was delineated in the midsagittal and midcoronal slices for each MRI scan.

All MRI scans were randomly divided into a training and test cohort at an 8:2 ratio after stratification by MRI location to ensure a balanced representation in both cohorts. A single annotator (I.B.) performed the delineation of pelvic structures and controlled 70 scans of the training cohort. These 70 scans were divided into three subsets, with each subset being controlled by one radiologist. The feedback received from these radiologists was then integrated into the remaining cases of both the training and test cohorts.

### Model development

Two three-dimensional convolutional neural networks (CNNs) were employed for training with a fivefold cross-validation for the coronal and sagittal directions, enabling automated segmentation of pelvic structures. We selected the state-of-the-art network architecture nnU-Net due to its automated pipeline that encompasses preprocessing to postprocessing. nnU-Net is an open-source model and has been successfully applied on many MRI-based anatomical sites [11], including small elongated neurovascular structures on prostate MRI [12]. Both nnU-Net models were trained for 1,000 epochs with a batch size of 2, utilizing a patch size of 24 × 256 × 256 and pooling operations of 2 along the first axis and 6 along the second and third axes.

### Pelvic measurements

**Fig. 1:**
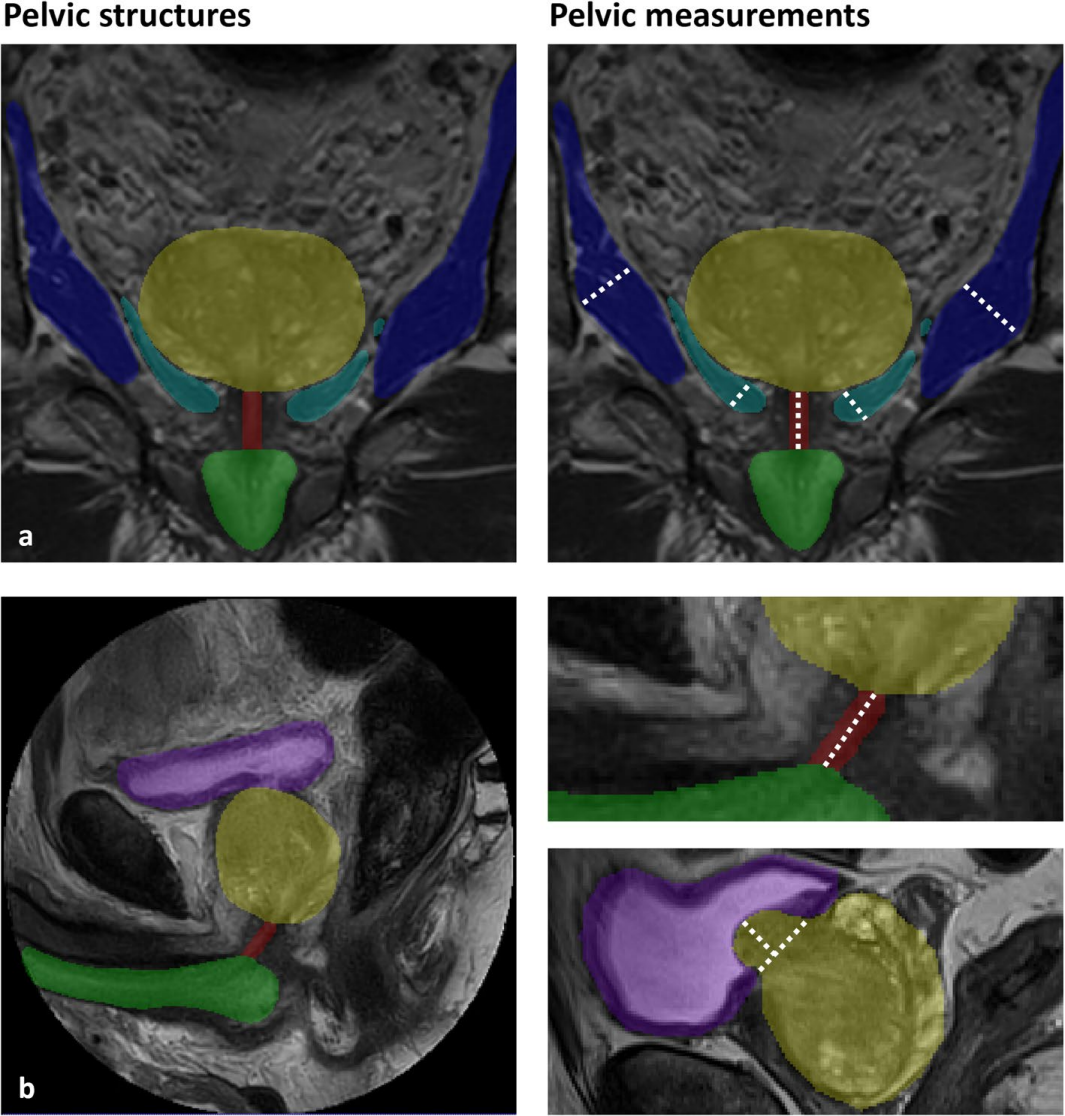
Pelvic structures and pelvic measurements in coronal direction (**a**) and sagittal direction (**b**) on T2-weighted images. Pelvic structures include the bladder (purple), corpus spongiosum (green), prostate (yellow), membranous urethra (red), obturator internus muscle (dark blue), and levator ani muscle (cyan)

Post-processing steps were performed to automate pelvic measurements on T2-weighted coronal and sagittal scans using a set of functions in Python 3.9, collecting the distances in millimeters (mm). The pelvic structures and measurements are shown in Fig. 1.

1. Coronal MUL: The largest segmentation in coronal plane was selected to determine the coronal MUL. We located the centroid of the segmentations in each axial slice and generated a line positioned at the center of the MUL for each axial slice to calculate the length in coronal direction. In cases where the membranous urethra was divided into two parts across adjacent slices, with one part connected to the prostate and the other part connected to the corpus spongiosum, both parts were considered for calculating the MUL.
2. Sagittal MUL: The largest segmentation in sagittal plane was selected to determine the sagittal MUL. The length was calculated with the same post-processing steps as for the coronal MUL.
3. IPPL: The length of the intravesical prostatic protrusion (IPPL) was determined by finding the intersection between the bladder and the prostate. This intersection was obtained by performing a one-pixel dilation of the bladder. A line was then formed connecting the outermost points of the intersection. The IPPL was computed as the maximum perpendicular distance between this line and the contour of the prostate.
4. LAM and OIM thickness: The maximum thickness of the left and right LAM and OIM was determined by compiling a list of the shortest distances between each point of the muscles’ centerline and its border in the coronal direction. The maximum distance from this list was selected and doubled to obtain the muscles' diameter.

### Model evaluation

The DL-generated segmentations of the pelvic structures were evaluated on the test cohort using volumetric Dice similarity coefficient (DSC), mean surface distances (MSD), and Hausdorff distances (HD95), utilizing the DeepMind Python package (https://github.com/deepmind/surface-distance). Volumetric DSC measures spatial overlap, MSD represent average distances, and HD95 capture maximum surface distances (95th percentile).

The pelvic MRI parameters (*i.e.*, coronal MUL, sagittal MUL, IPPL, OIM thickness and LAM thickness) were evaluated on the test cohort in two sessions by three abdominal radiologists (F.W., E.H., C.T.N.). The first session comprised manual measurements on raw MRI images in coronal and sagittal direction and the second session involved manual measurements on MRI images with DL-generated segmentations of pelvic structures in ITK-SNAP (version 3.6.0; http://www.itksnap.org). The evaluation of the second session was conducted at least seven days after the first evaluation, and the patient order was mixed to avoid recall bias. The radiologists were blinded to patients’ clinical data.

**Fig. 2:**
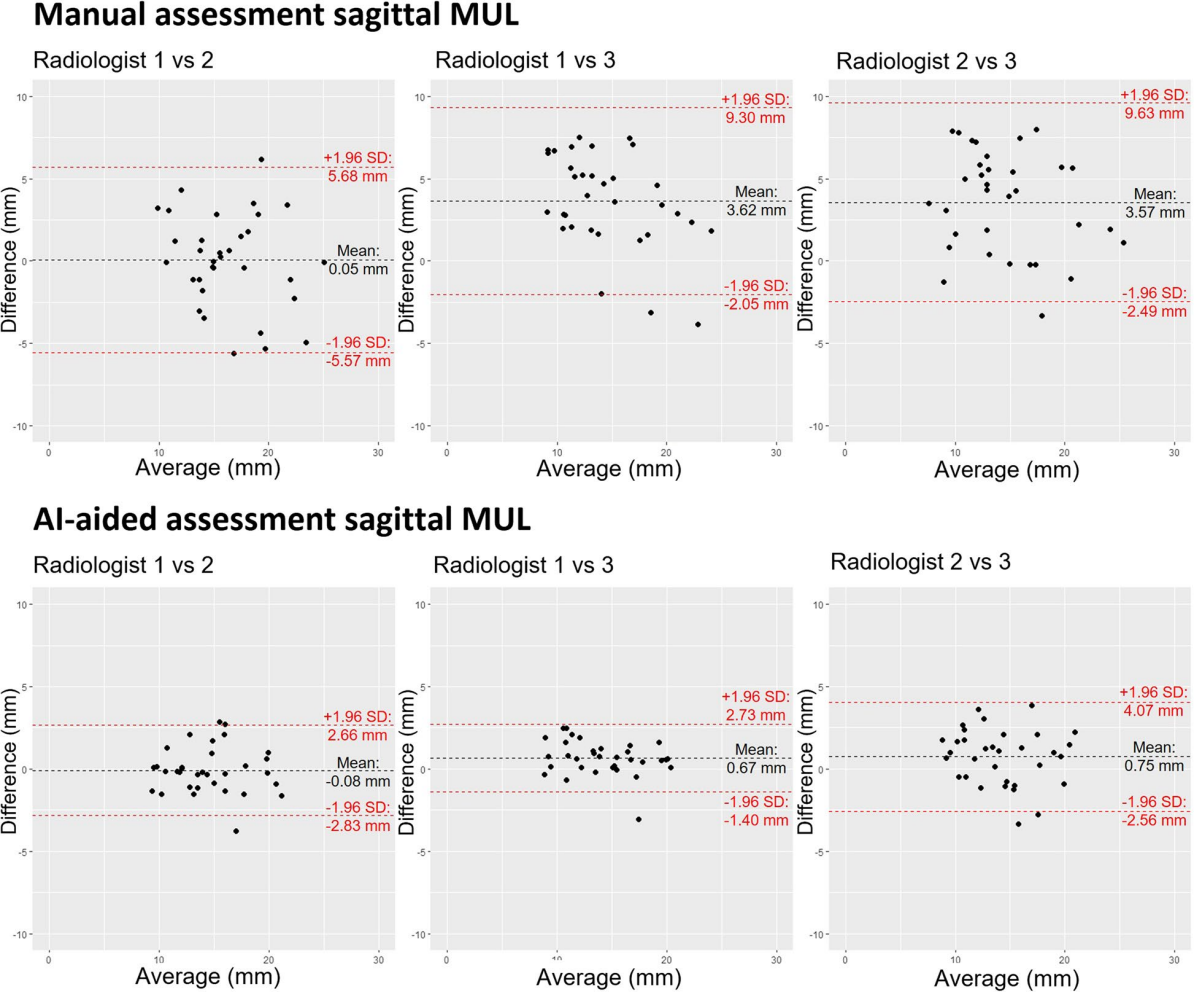
Bland–Altman plots of measured membranous urethral length (MUL) in the sagittal direction for manual assessment and artificial intelligence (AI)-aided assessment. The black dashed line represents the mean difference and the red dashed lines represent the upper and lower 95% limits of agreement

The absolute interobserver agreement for each measurement was determined by applying a two-way random effect analysis with the intraclass correlation coefficient (ICC). ICC values ≤ 0 indicate no agreement, 0.01–0.20 as none to slight agreement, 0.21–0.40 as fair agreement, 0.41–0.60 as moderate agreement, 0.61–0.80 as substantial agreement, and 0.81–1.00 as almost perfect agreement [13]. The interobserver agreement for each measurement was visualized in Bland–Altman plots between the manual and the AI-aided measurements (Fig. 2). Non-normally distributed data were presented as median with interquartile range (IQR). Wilcoxon-signed rank tests were conducted and *p* < 0.050 was considered statistically significant.

## Results

### Segmentation performance

Table 3 presents the DL segmentation performances in the coronal and sagittal planes. The median DSC values ranged from 0.85 to 0.97 for coronal structures and from 0.87 to 0.98 for sagittal structures. The highest DSC value was observed for the bladder (DSC 0.98; IQR 0.97–0.98), while the lowest DSC value was found for the membranous urethra (DSC 0.85; IQR 0.76–0.92) in the coronal direction. The median MSD values ranged from 0.10 to 0.31 mm, with the lowest value observed for the membranous urethra in the sagittal direction (MSD 0.10 mm; IQR 0.05–0.14). Additionally, the DL network prediction time for the sagittal test cohort was 2 min and 40 s (approximately 5 s per scan) and for the coronal test cohort 3 min and 5 s (approximately 6 s per scan).

**Table 3:** Deep learning segmentation performances

	DSC	MSD [mm]	HD95 [mm]	Volume [cc]
Coronal structures				
Prostate	0.97 (0.96–0.97)	0.22 (0.16–0.32)	1.25 (0.87–1.75)	40.93 (36.30–54.02)
Urethra	0.85 (0.76–0.92)	0.14 (0.07–0.32)	1.25 (0.60–3.01)	0.20 (0.17–0.24)
Corpus spongiosum	0.96 (0.96–0.97)	0.10 (0.09–0.12)	0.60 (0.42–0.63)	9.73 (8.04–12.47)
OIM	0.95 (0.92–0.96)	0.25 (0.15–0.36)	1.25 (0.63–3.00)	38.17 (33.80–43.21)
LAM	0.90 (0.86–0.91)	0.24 (0.17–0.33)	2.99 (0.88–3.00)	5.92 (5.08–6.83)
Sagittal structures				
Bladder	0.98 (0.97–0.98)	0.15 (0.11–0.23)	0.63 (0.63–1.20)	100.64 (73.47–148.26)
Prostate	0.96 (0.94–0.97)	0.31 (0.22–0.47)	1.25 (1.12–1.90)	37.23 (32.52–46.61)
Urethra	0.87 (0.77–0.90)	0.10 (0.05–0.14)	0.82 (0.63–1.88)	0.21 (0.18–0.24)
Corpus spongiosum	0.94 (0.90–0.95)	0.22 (0.16–0.31)	1.88 (0.88–3.00)	10.84 (9.57–13.04)

For the LAM and IOM: left and right are combined. Data are expressed as median (interquartile range). *DSC* Dice similarity coefficient, *MSD* Mean surface distance, *HD95* 95% boundary Hausdorff distance, *LAM* Levator ani muscle, *OIM* Obturator internus muscle

### Pelvic measurements

The pelvic measurements are presented in Table 4 for the three different measurement approaches: manual, AI-aided, and automated. The automated approach resulted in significantly higher values for the IPPL, LAM thickness, and OIM thickness (*p* < 0.001) and showed comparable values for the coronal MUL (*p* = 0.091) and sagittal MUL (*p* = 0.606) in comparison to the AI-aided approach. Sagittal MUL was highest for the manual approach (14.94 mm; IQR 12.08–17.73), followed by the automated approach (14.32 mm; IQR 12.50–17.12), and the AI-aided approach (14.04; IQR 11.63–16.97). Coronal MUL measurements were found to be lower, with a median value of 13.78 mm for the manual and AI-aided approach, and 14.06 for the automated approach. The median LAM thickness ranged from 8.56 to 11.14 mm, and the OIM thickness ranged from 17.92 to 19.77 mm, with the automated approach yielding the highest median values for both measurements.

**Table 4:** Comparison of pelvic measurements on T2-weighted MRI: manual, AI-aided and automated workflows with intraclass correlation coefficients

Measurements	Manual [mm]	AI-aided [mm]	Automated [mm]	ICC (Manual)	ICC (AI-aided)
Coronal MUL	13.78 (11.55–16.10)	13.78 (12.20–15.67)	14.06 (13.12–16.41)	0.69 (0.51–0.82)	0.90 (0.82–0.94)
Sagittal MUL	14.94 (12.08–17.73)	14.04 (11.63–16.97)	14.32 (12.50–17.12)	0.64 (0.28–0.83)	0.91 (0.84–0.95)
IPPL	0.00 (0.00–0.00)	0.00 (0.00–0.00)	2.40 (1.09–3.26)	0.88 (0.81–0.94)	0.86 (0.77–0.93)
LAM thickness	8.75 (7.27–10.08)	8.56 (7.35–10.05)	11.14 (10.18–12.31)	0.40 (0.05–0.66)	0.61 (0.31–0.78)
OIM thickness	18.05 (16.23–20.28)	17.92 (15.96–20.31)	19.77 (17.93–21.93)	0.74 (0.42–0.87)	0.86 (0.75–0.92)

For the LAM and OIM: left and right are combined. Data are expressed as median (interquartile range). *AI* Artificial intelligence, *ICC* Intraclass correlation coefficient, *MUL* Membranous urethral length, *IPPL* Intravesical prostatic protrusion length, *LAM* Levator ani muscle, *OIM* Obturator internus muscle

### Interobserver agreement

The ICC values ranged from 0.40 to 0.88 for the manual approach and from 0.61 to 0.91 for the AI-aided approach (Table 4). The highest interobserver agreement was observed for sagittal MUL (ICC 0.91, 95% CI 0.84–0.95) with the AI-aided approach, indicating almost perfect agreement. The manual approach showed substantial agreement (ICC 0.64, 95% CI 0.28–0.83). The lowest interobserver agreement was found for LAM thickness, with the manual approach demonstrating moderate agreement (ICC 0.40, 95% CI 0.05–0.66) and the AI-aided approach showing substantial agreement (ICC 0.61, 95% CI 0.31–0.78). A Bland–Altman plot of sagittal MUL is presented in Fig. 2, showing the median difference between the manual and AI-aided approaches. The Bland–Altman plots of the other pelvic measurements are shown in Supplementary Figs. 1–4.

## Discussion

To our knowledge, this is the first study that developed and evaluated an automated workflow to measure pelvic floor parameters associated with post-prostatectomy incontinence. The implementation of DL-generated segmentations of pelvic structures resulted in higher interobserver agreement among radiologists. Both coronal and sagittal MUL measurements demonstrated improved interobserver agreement, with the coronal MUL increasing from an ICC value of 0.69 (95% CI 0.51–0.82) to 0.90 (95% CI 0.82–0.94) and the sagittal MUL improving from 0.64 (95% CI 0.28–0.83) to 0.91 (95% CI 0.84–0.95), indicating a shift from substantial to almost perfect agreement. Veerman et al. [7] reported a comparable ICC value of 0.63 (95% CI 0.28–0.81) for the sagittal MUL measurements on raw images but achieved an improved ICC value of 0.84 (95% CI 0.66–0.91) with the implementation of consistent anatomical definitions. In our study, DL-generated segmentations resulted in a higher ICC value of 0.91 (95% CI 0.84–0.95), demonstrating the potential of AI to further improve consistency in the measurements.

Additionally, the use of DL-generated segmentations improved interobserver agreement for the musculoskeletal structures. The OIM thickness had an ICC value of 0.74 (95% CI 0.42–0.87) for the manual assessment and 0.86 (95% CI 0.75–0.92) for the AI-aided assessment, indicating an improvement from substantial agreement to almost perfect agreement. The LAM thickness showed moderate agreement for the manual approach (ICC 0.40; 95% CI 0.05–0.66) and improved to substantial agreement for the AI-aided approach (ICC 0.61; 95% CI 0.31–0.78). However, IPPL measurements demonstrated no improvement with DL-generated segmentations, with ICC values of 0.88 (95% CI 0.81–0.94) and 0.86 (95% CI 0.77–0.93) for the manual and AI-aided approach, respectively.

In comparison with the manual and AI-aided approaches, the automated approach yielded significantly higher values for the LAM thickness, OIM thickness, and the IPPL. The higher automated muscle thickness could be explained by the fact that the segmentations around the muscle borders were slightly larger in some cases. In addition, variations in muscle diameters may be attributed to the computer’s capability to accurately compute the maximum diameter, while variations in angle or slice positioning during manual measurements by radiologists may affect the measured diameter. In future studies, it may be beneficial to present the computed thickness to radiologists, enabling them to verify the measurements and potentially minimize the need for manual measurements. The automated IPPL measurements also demonstrated higher values compared to the manual and AI-aided measurements, often resulting in an overestimation of the protrusion. Consequently, manual assessments of DL-generated segmentations remain important, especially in cases where segmentation errors may arise.

The automated sagittal and coronal MUL measurements were not statistically different from the AI-aided measurements. The automated sagittal MUL measurements were 14.32 mm, the AI-aided measurements were 14.04 mm, and the manual measurements were 14.94 mm. The larger manual MUL measurements can be attributed to the different positioning of the superior point of the membranous urethra. During the first session, two radiologists defined the superior point in some patients differently, placing it more superiorly where the urethra was still visible in the caudal portion of the prostate apex. This superior point may align more closely with the surgical section during RARP, where urologists aim to preserve the membranous urethra for the vesicourethral anastomosis [14], except in cases involving apical tumors. In the second session, the superior point was consistently positioned at the inferior border of the prostate due to the DL-generated segmentations, which contributed to increased consistency as indicated by the AI-aided measurements. This definition of the MUL measurement was comparable to previous studies [3, 6, 7].

The MUL measurements in our study were comparable with Kim et al. [3], who reported mean values of 14.6 mm in the coronal direction and 14.2 mm in the sagittal direction. Veerman et al. [7] found higher MUL measurements of 17 mm, which could potentially be attributed to the inclusion of patients with higher MUL values since the introduction of a risk prediction model of urinary incontinence in their institution [15]. LAM measurements were comparable to the findings of Sadahira et al. [16] but higher than the measurements reported by Muñoz-Calahorro et al. [6], which ranged between 4.22 and 6.87 mm. These differences in measurements could be attributed to variances in anatomical definition, as Muñoz-Calahorro et al. [6] measured the thickness before the insertion of the muscle puborectalis fibers, while we measured the largest thickness regardless of its location.

More consistent pelvic measurements are especially clinically relevant when including them in urinary incontinence prediction models for patients undergoing RARP. Multiple studies have already created prediction models for continence outcome after RARP and incorporated patient-related variables and MRI-based variables like the MUL [10, 17–19]. Standardized measurements are essential since the continence prediction tool described in Tillier et al. [10] demonstrates that a 1-mm increase in the MUL corresponds to an average increase of 8.1 percentage points in the chance of continence recovery after six months of RARP [7]. These findings can influence the treatment choices of PCa patients. Therefore, standardizing prostate MRI measurements using DL is an unmet need for continence prediction models. Additionally, DL-generated segmentations provide an opportunity to assess additional geometric variables, including shape-based features, surface areas, and volume-based features.

This study had some limitations. First, not all training cases were verified by three radiologists but a subset of 70 patients was evaluated and the feedback of the three different radiologists was used. Second, the manual assessment of the pelvic measurements was performed in sagittal and coronal plane. It was not possible to visualize the axial, coronal, and sagittal planes simultaneously. The visualization of the prostate in three directions would have enhanced the ease of evaluating MUL measurements. Third, external validation of our DL model and automated measurements in other centers is essential because only a part of the T2-weighted images adhered to the PI-RADSv2 MRI acquisition parameter guideline [20]. We believe that our DL model has the potential for generalizability, as it has been trained using data from five regional hospitals with various magnetic field strengths, vendors, and sequence protocols. Additional training cases with variations in anatomy, such as more patients with IPP and patients with hip prostheses, could contribute to improving the generalization ability of our DL model. Finally, the clinical impact of AI-aided assessment of pelvic parameters could not be retrospectively assessed. Future studies are needed to evaluate the relationship between objective pelvic measurements and postoperative outcomes.

In conclusion, our study demonstrates the potential of deep learning (DL)-generated segmentations to improve interobserver agreement of pelvic measurements among radiologists. The membranous urethral length (MUL) measurement improved from substantial agreement to almost perfect agreement among radiologists with DL-generated segmentations of pelvic structures. Standardized pelvic measurements can be incorporated into urinary incontinence prediction models for patients undergoing RARP.

### Supplementary Information

**Additional file 1:** **Supplementary Figure 1.** Bland-Altman plots of measured membranous urethral length (MUL) in the coronal direction for manual assessment and AI-aided assessment. **Supplementary Figure 2.** Bland-Altman plots of measured intravesical prostatic protrusion length (IPPL) in the sagittal direction for manual assessment and AI-aided assessment. **Supplementary Figure 3.** Bland-Altman plots of measured obturator internus muscle (OIM) thickness in the coronal direction for manual assessment and AI-aided assessment. **Supplementary Figure 4.** Bland-Altman plots of measured levator ani muscle (LAM) thickness in the coronal direction for manual assessment and AI-aided assessment.

## Data Availability

The datasets used and/or analyzed during the current study are available from the corresponding author on reasonable request.

## Abbreviations

AI: Artificial intelligence
CI: Confidence interval
DL: Deep learning
DSC: Dice similarity coefficient
ICC: Intraclass correlation coefficient
IPPL: Intravesical prostatic protrusion length
IQR: Interquartile range
LAM: Levator ani muscle
MRI: Magnetic resonance imaging
MSD: Mean surface distances
MUL: Membranous urethral length
OIM: Obturator internus muscle
PCa: Prostate cancer
RARP: Robot-assisted radical prostatectomy
