# Supplementary material for: Automated pelvic MRI measurements associated with urinary incontinence for prostate cancer patients undergoing radical prostatectomy
Source: Eur Radiol Exp. 2024 Jan 2;8:1. doi: 10.1186/s41747-023-00402-4 (PMC10761662; doi:10.1186/s41747-023-00402-4)
Supplement: Supplementary file 1 — Additional file 1: Supplementary Figure 1. Bland-Altman plots of measured membranous urethral length (MUL) in the coronal direction for manual assessment and AI-aided assessment. Supplementary Figure 2. Bland-Altman plots of measured intravesical prostatic protrusion length (IPPL) in the sagittal direction for manual assessment and AI-aided assessment. Supplementary Figure 3. Bland-Altman plots of measured obturator internus muscle (OIM) thickness in the coronal direction for manual assessment and AI-aided assessment. Supplementary Figure 4. Bland-Altman plots of measured levator ani muscle (LAM) thickness in the coronal direction for manual assessment and AI-aided assessment. [file 41747_2023_402_MOESM1_ESM.docx]

**Automated pelvic MRI measurements associated with urinary incontinence for prostate cancer patients undergoing radical prostatectomy**

**ELECTRONIC SUPPLEMENTARY MATERIAL**

**Manual assessment coronal MUL**

**AI-aided assessment coronal MUL**


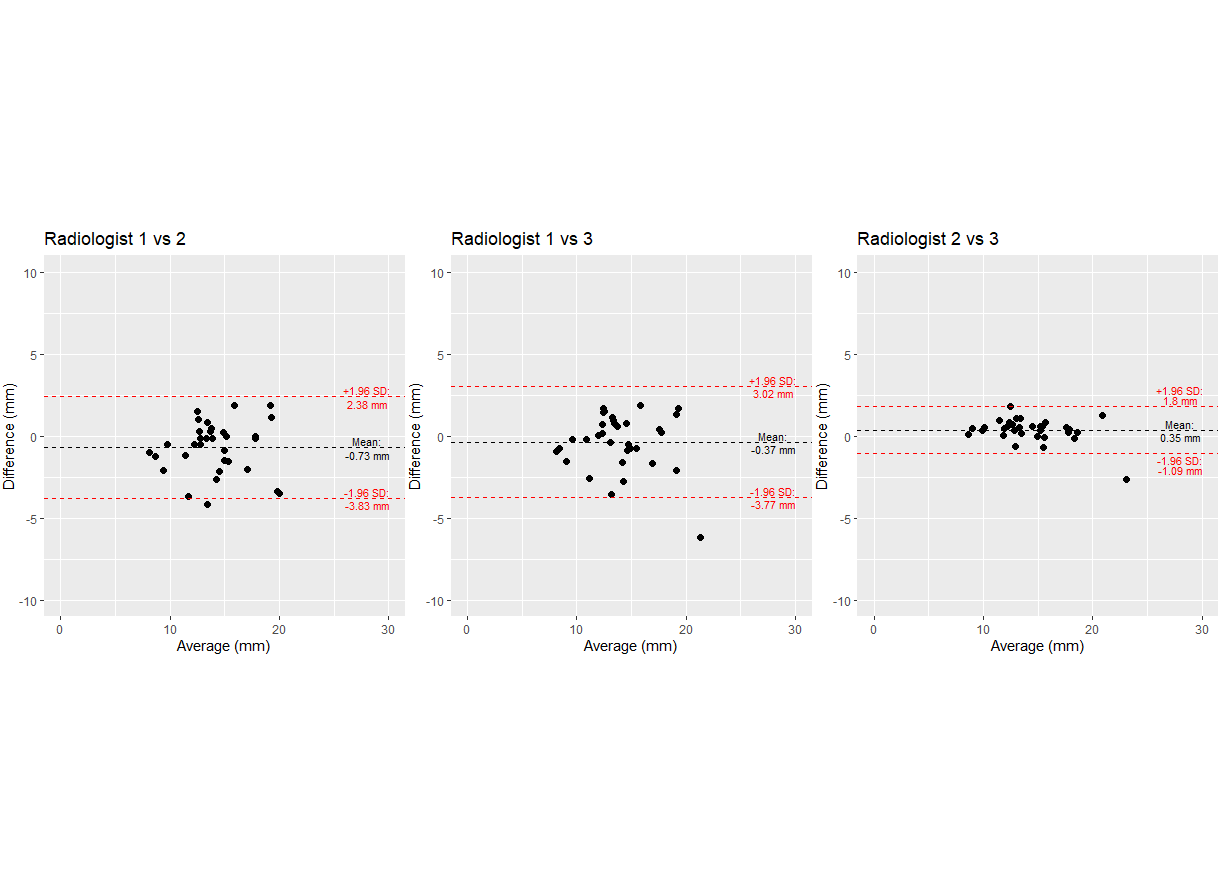

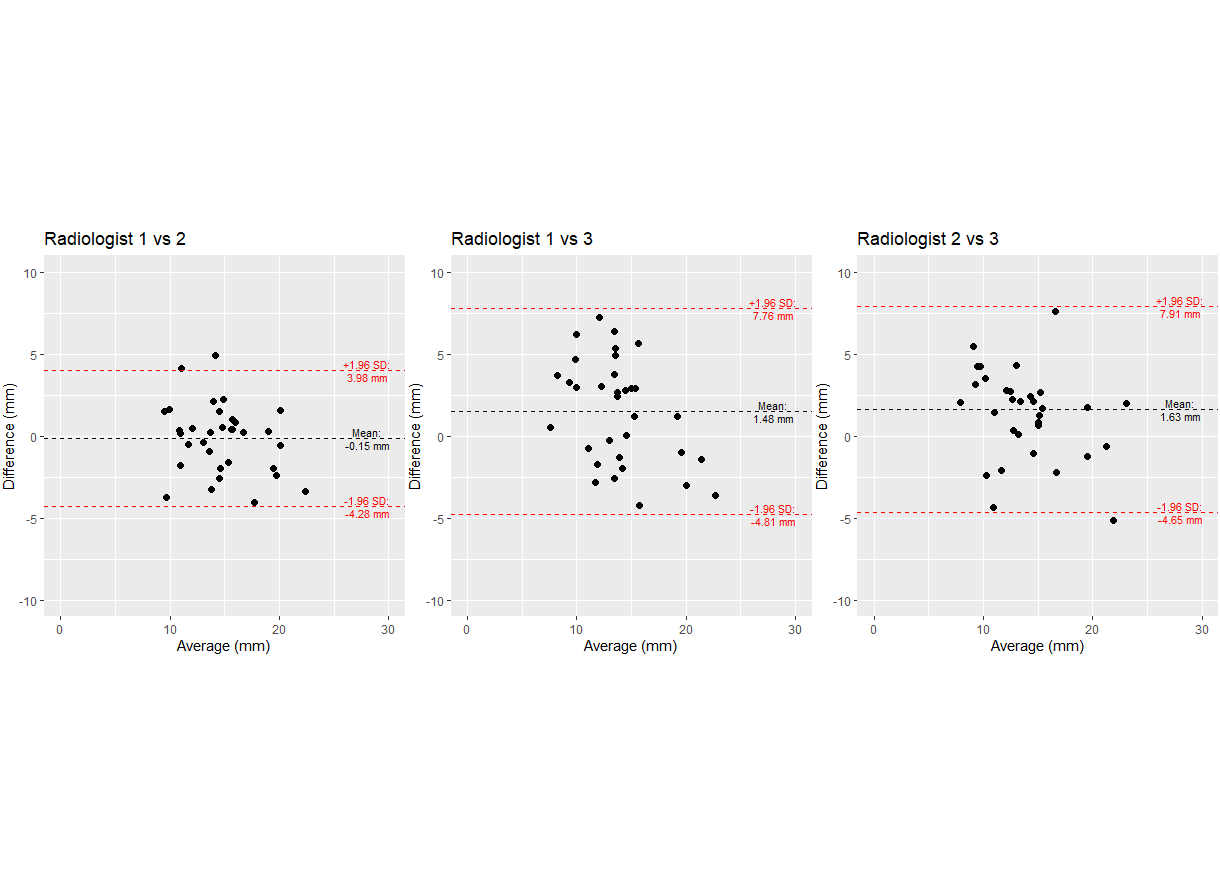


**Supplementary figure 1.** Bland-Altman plots of measured membranous urethral length (MUL) in the coronal direction for manual assessment and AI-aided assessment. The black dashed line represents the mean difference and the red dashed lines represent the upper and lower 95% control limits.

**Manual assessment IPPL**

**AI-aided assessment IPPL**


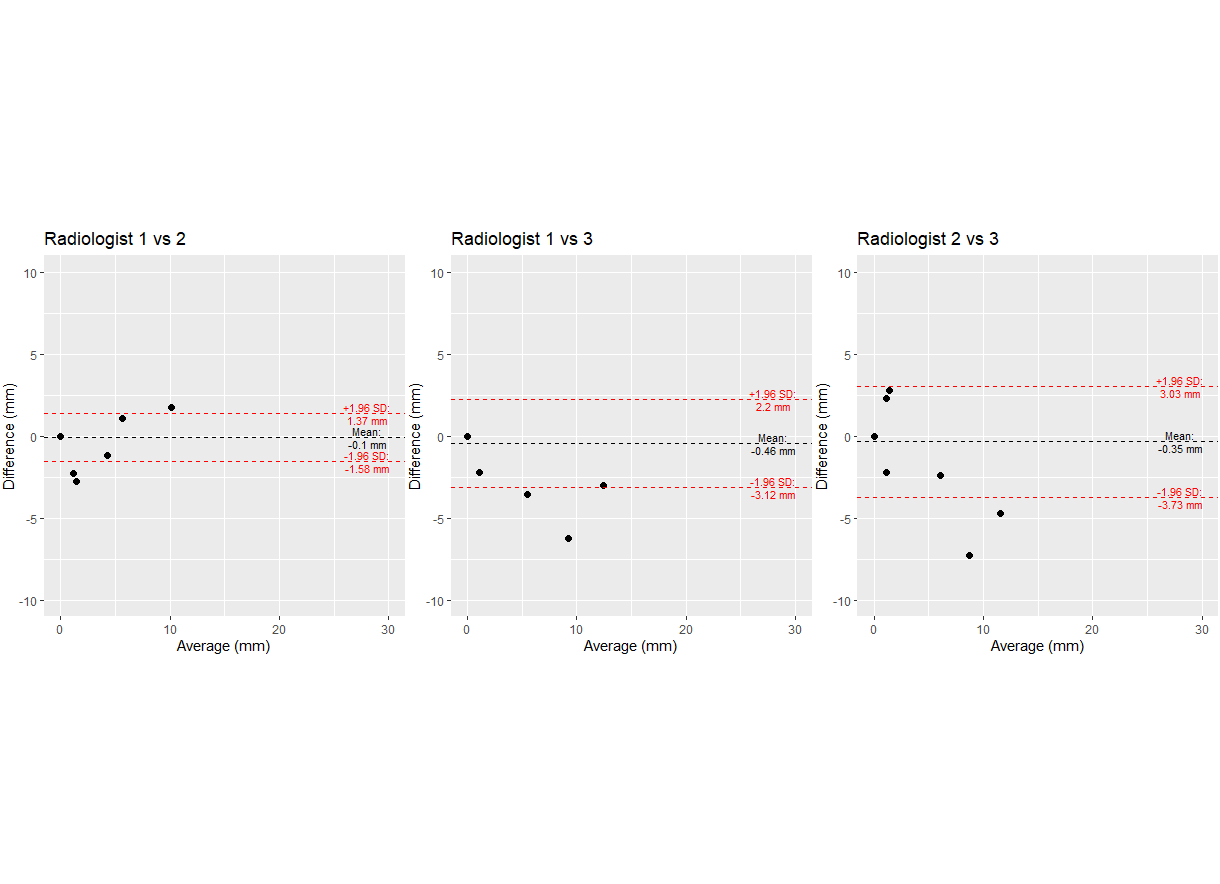

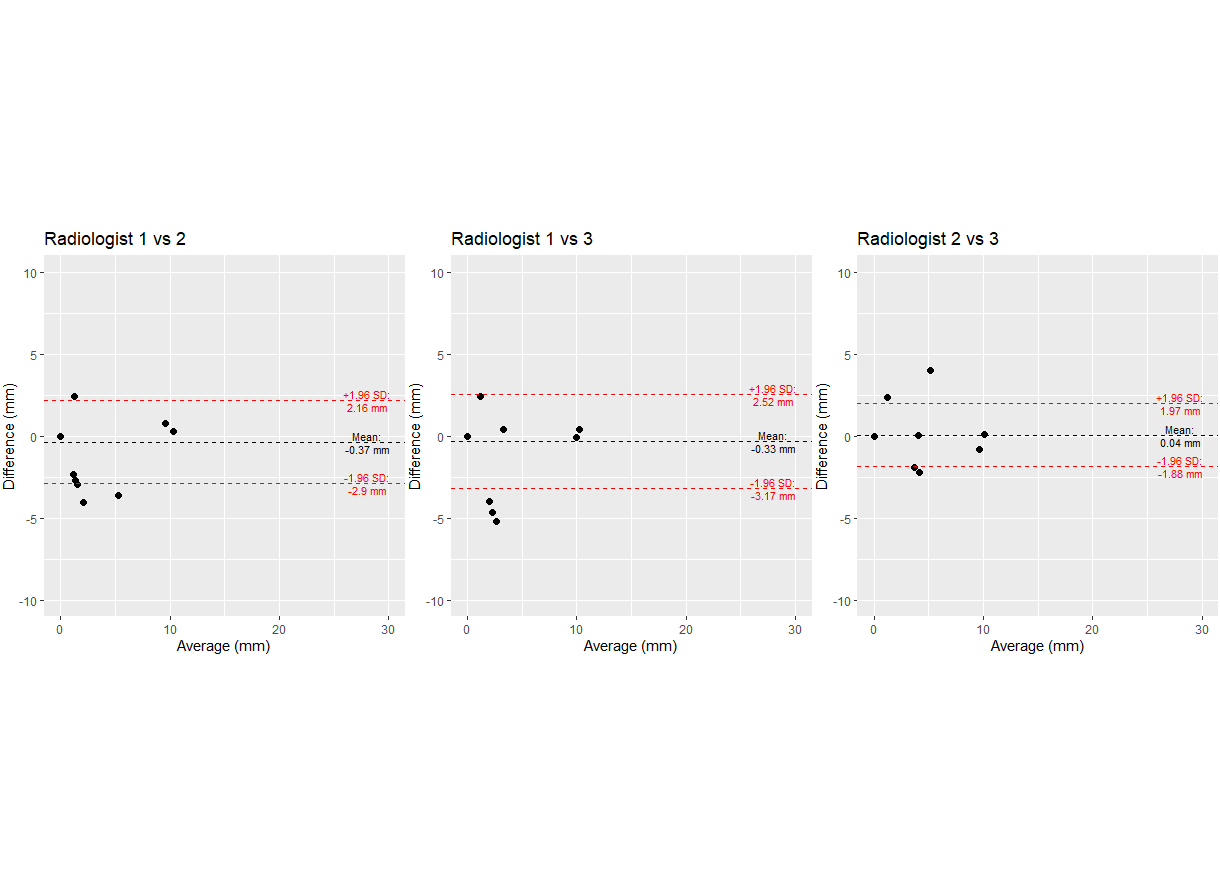


**Supplementary figure 2.** Bland-Altman plots of measured intravesical prostatic protrusion length (IPPL) in the sagittal direction for manual assessment and AI-aided assessment. The black dashed line represents the mean difference and the red dashed lines represent the upper and lower 95% control limits.

**Manual assessment OIM thickness**

**AI-aided assessment OIM thickness**


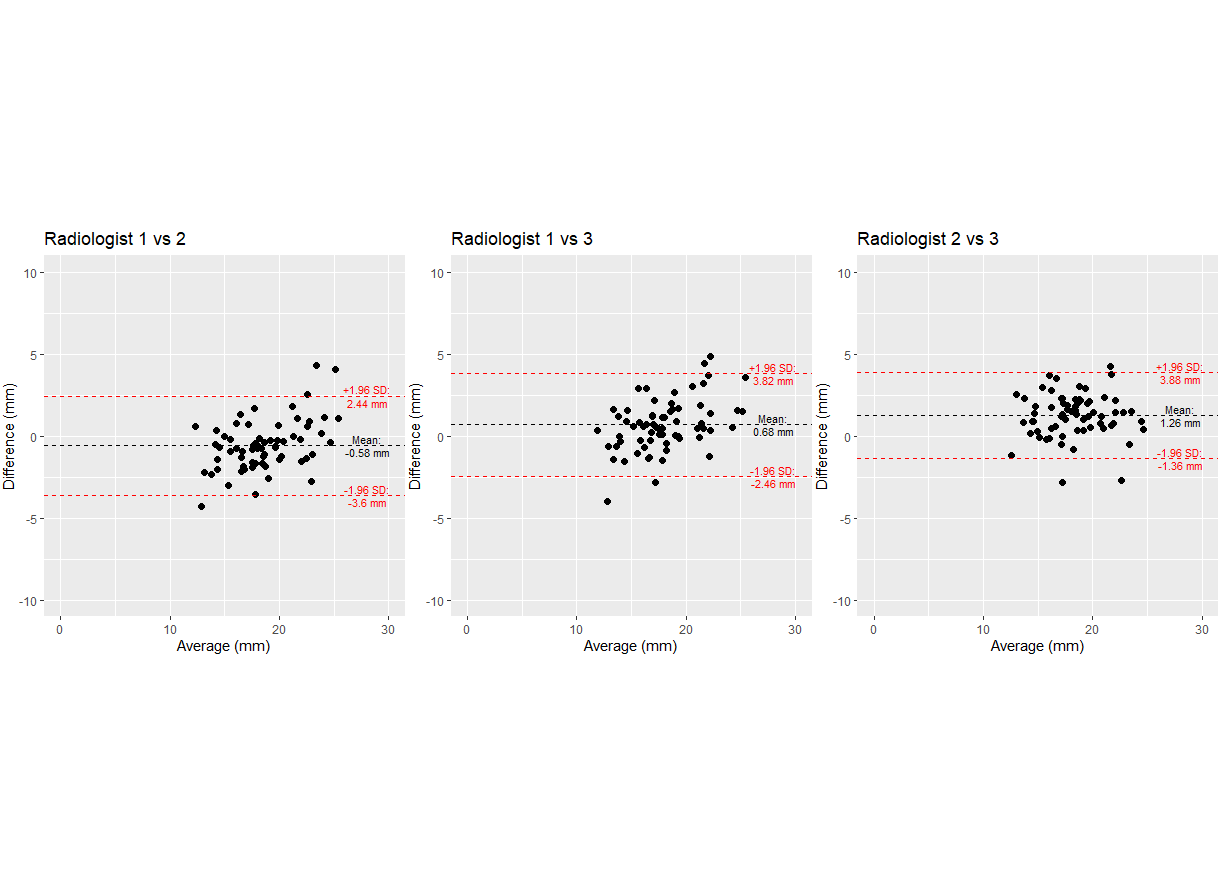

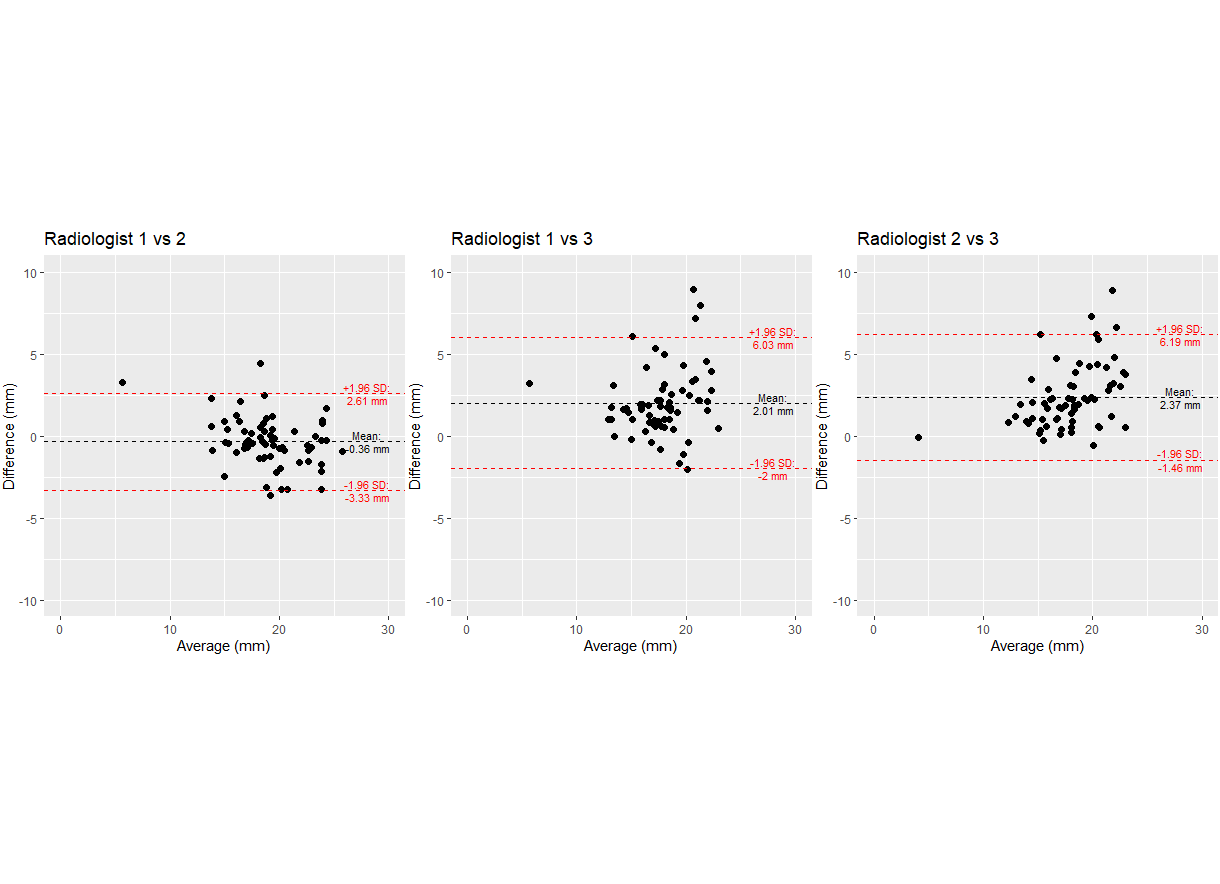


**Supplementary figure 3.** Bland-Altman plots of measured obturator internus muscle (OIM) thickness in the coronal direction for manual assessment and AI-aided assessment. The black dashed line represents the mean difference and the red dashed lines represent the upper and lower 95% control limits.

**Manual assessment LAM thickness**

**AI-aided assessment LAM thickness**


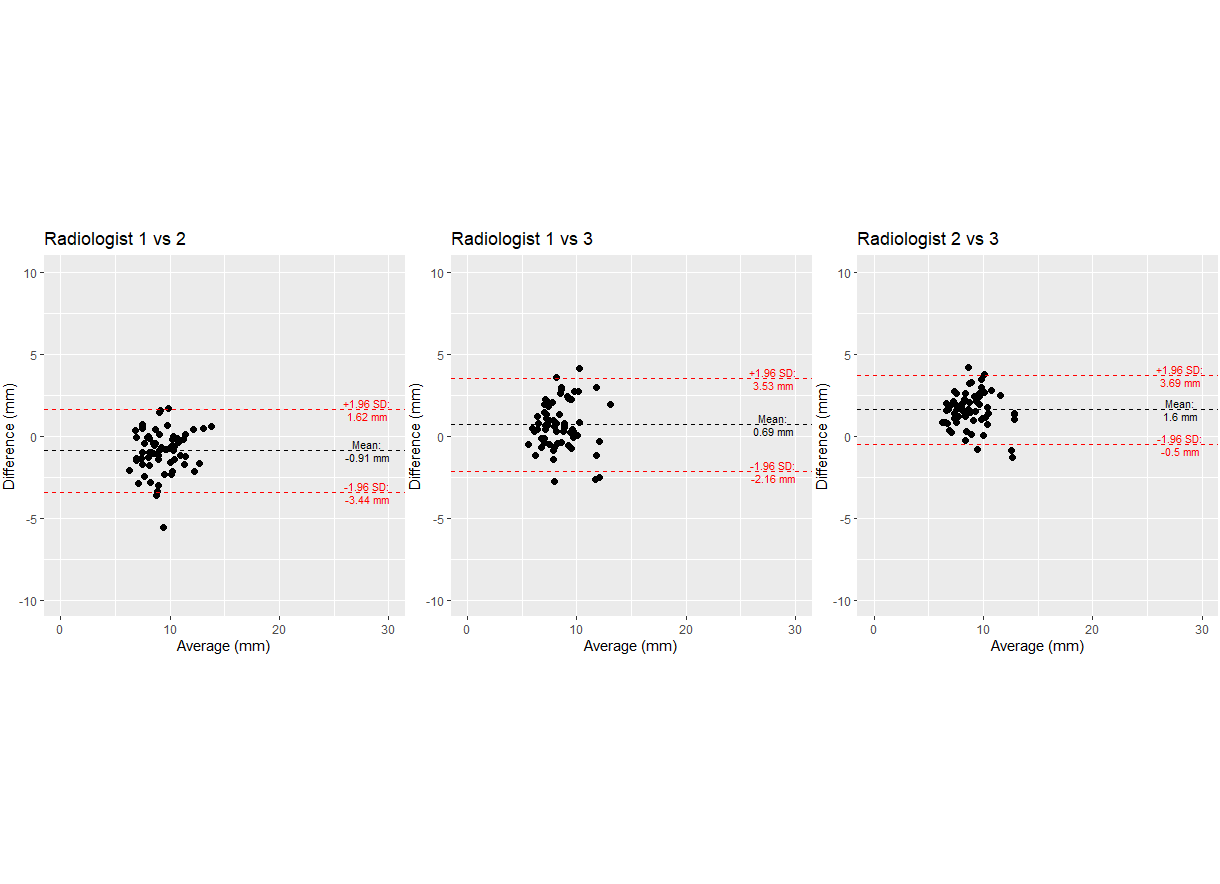

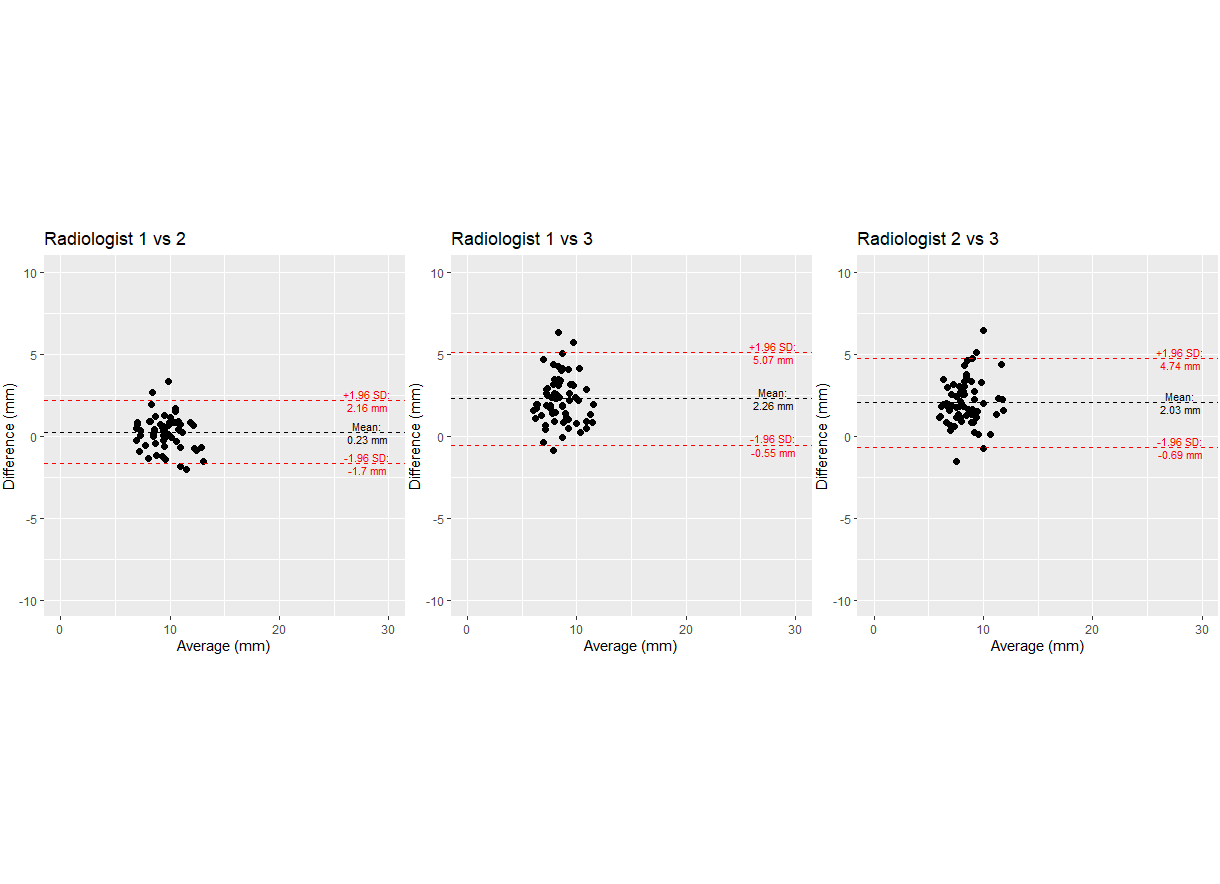


**Supplementary figure 4.** Bland-Altman plots of measured levator ani muscle (LAM) thickness in the coronal direction for manual assessment and AI-aided assessment. The black dashed line represents the mean difference and the red dashed lines represent the upper and lower 95% control limits.
